# The applicability of normalisation process theory to speech and language therapy: a review of qualitative research on a speech and language intervention

**DOI:** 10.1186/1748-5908-6-95

**Published:** 2011-08-12

**Authors:** Deborah M James

**Affiliations:** 1 National Institute of Health Research Biomedical Research Unit in Hearing, 113 The Ropewalk, Nottingham, United Kingdom, NG1 5DU

## Abstract

**Background:**

The Bercow review found a high level of public dissatisfaction with speech and language services for children. Children with speech, language, and communication needs (SLCN) often have chronic complex conditions that require provision from health, education, and community services. Speech and language therapists are a small group of Allied Health Professionals with a specialist skill-set that equips them to work with children with SLCN. They work within and across the diverse range of public service providers. The aim of this review was to explore the applicability of Normalisation Process Theory (NPT) to the case of speech and language therapy.

**Methods:**

A review of qualitative research on a successfully embedded speech and language therapy intervention was undertaken to test the applicability of NPT. The review focused on two of the collective action elements of NPT (relational integration and interaction workability) using all previously published qualitative data from both parents and practitioners' perspectives on the intervention.

**Results:**

The synthesis of the data based on the Normalisation Process Model (NPM) uncovered strengths in the interpersonal processes between the practitioners and parents, and weaknesses in how the accountability of the intervention is distributed in the health system.

**Conclusions:**

The analysis based on the NPM uncovered interpersonal processes between the practitioners and parents that were likely to have given rise to successful implementation of the intervention. In previous qualitative research on this intervention where the Medical Research Council's guidance on developing a design for a complex intervention had been used as a framework, the interpersonal work within the intervention had emerged as a barrier to implementation of the intervention. It is suggested that the design of services for children and families needs to extend beyond the consideration of benefits and barriers to embrace the social processes that appear to afford success in embedding innovation in healthcare.

## Background

In his review of the services for children with speech, language, and communication needs (SLCN) in England and Wales, Bercow [1] said that, 'The requirements of children and young people with SLCN and their families will be met when, and only when, appropriate services to support them, across the age range and spectrum of need, are designed and delivered in a way that is accessible to them.' Over one-half of the 1,000 families who participated in the consultation said that speech and language therapy services were poor. Whilst families indicated that improvements in services could come from enhanced resourcing, their evidence also showed that there is an imperative for change in the design and delivery of speech and language therapy. In response, the Department for Children Schools and Families published an action plan for improvement in public services [2] that committed to a series of initiatives, many funded, to improve services for children with speech language and communication needs. If services are going to change to be more family-centred, then we need to know more about what families want from services at different points in their trajectory of service involvement [3]. Bercow placed a high priority on early identification and early intervention for children with SLCN. However, Lindsay *et al.*' [4] primary qualitative research showed that wide variation exists across health and educational providers with regards to the practice of identification of children with SLCN as well as the provision of services to meet their needs. It is an opportune time to consider the complex context in which speech and language interventions are delivered to explore: how intervention research should be designed so that interventions can be integrated across and within the diverse public service delivery context; and how interventions can be designed to better meet the specific needs and expectations of the families themselves.

To date, there are only a handful of studies on the parental perspective on speech and language therapy [5-13], and only three of these studies have used the type of qualitative methodology that is needed to explore the parents' frame of reference [6,7,11]. Given the priority for early intervention, the focus on the transition into speech and language therapy is a good place to start to deepen understanding of the perspectives of the main participants. The discussions at transition points are considered to be opportune times to engage all stakeholders as active participants to help keep the child and family at the centre of the healthcare system [14]. Getting active participation of patients in healthcare is known to be associated with higher treatment compliance [15]. The achievement of active participation is crafted in large part within the conversational encounters between clinicians and patients [16]. There has been limited exploration of these concepts in speech and language therapy, but the results of the Bercow review suggest that these are priority areas for speech and language therapy research and practice.

Speech and language therapy interventions are good examples of complex interventions. They do not typically involve drug or surgical interventions; rather, the interventions are most often behaviourally based and delivered through discourse between the practitioner and the patient. Second, the allied health professions are small groups within healthcare systems, and this means that they usually work in distributed teams within healthcare services. Finally, the evidence base for speech and language therapy interventions is still developing. A systematic review of speech and language therapy for children found 25 randomised controlled trials since the 1960s [17]. Whilst the impact of speech and language therapy for children with some types of speech/language problems was concluded to be positive, there was high heterogeneity across the studies included in the review with subsequent impact on the confidence intervals of the effect sizes from the meta-analysis. The UK speech and language profession is considered to have a relatively strong research base in terms of quality of publications and percentage of international published contributions in biomedical scientific journals [18], however, we can see from the work of Law *et al. *that the evidence base of randomised controlled trails is small, and this has an impact on the nature of the conclusions that can be drawn from meta-analyses. Thus, we have a situation where the profession is comparatively research-engaged, but the evidence base for the interventions delivered by the profession is weak. Public dissatisfaction with services is well documented. With new post-Bercow funding for intervention research, it is especially important that the potential implementation of new interventions is explored at an early stage so that, if found to be efficacious, new interventions can be quickly embedded within existing practice.

The complex nature of the interventions and the diverse delivery conditions of those interventions across a range of public services provides a challenging context in which to design new interventions that can be embedded in practice for patient benefit. Murray *et al. *[19] suggested that Normalisation Process Theory (NPT) provides a framework that can be used to design and evaluate complex interventions to improve potentiality for implementation of research interventions in practice.

### Normalisation process theory

The NPT [20] grew out of the Normalisation Process Model (NPM) [21] and May's interest in understanding the work that is done by individuals and collectives of people to get innovation normalised as part of everyday practice in the context of healthcare delivery. An original concept of the NPM concerned the way healthcare interventions are co-constructed between different agents in the intervention (patient, provider, and other healthcare workers). The role of collective action was characterised as one of four main types of work in the subsequent NPT. The NPM offers a set of explanatory propositions of how different internal intervention elements and external intervention elements support the embedding of the intervention in practice. The model was been built on qualitative data on the introduction of new technologies in healthcare and the management of chronic illness in primary care in the UK. It has four main categories: professional-patient relationships; new modalities for delivering care; social construction and production of evidence; and social organisation of clinical work. According to the model, interventions will be likely to be embedded if they afford a high level of flexibility in the internal elements of the intervention. This includes elements such as establishing the meaning of the intervention, agreeing the way in which the intervention will be delivered, and evaluating the effectiveness of the intervention between the participants. According to the NPM, interventions that develop evaluation protocols based on how all the participants attribute meaning to the intervention will tend to be more successful in their ability to become embedded in practice.

### Applying Normalisation Process Model to speech and language therapy

Despite the influence of the Medical Research Council's guidance for designing complex interventions [22], there is an acknowledgement that results from intervention research, specifically randomised controlled trials, often fail to provide useful information [19]. Campbell *et al. *attribute this to a lack of theoretically motivated groundwork in the initial stages of the intervention design [23]. They highlight the opportunity to draw on health psychology and social theory to fully explore and model the multiple and complex mechanisms of change in intervention design. If the time-limited opportunity for more intervention research in speech and language therapy is to have maximum effect in public services, then raising the profile of the role of health psychology and social theory to the research designers in the field is warranted. There is a call for the application of more social theory in speech and language therapy research [24], but there is currently a very limited amount of qualitative research in the field.

### Research question

The primary aim of this study was to test the applicability of the propositions on the role of collaborative work laid out in the NPM and NPT to the context of speech and language therapy so that, if found to be applicable, the NPT could be used to inform the design of new intervention research in the field. Specifically, I set out to test the theory according to the requirements a theory as set out by May *et al. *[25]. I wanted to find out: whether the definitions as described within the original version of the NPM could be applied to a new data set based on a synthesis of qualitative research from previously published research on a successfully embedded speech and language intervention (see below); whether the application of the model could uncover new understanding of how the interpersonal work done by the participants of the intervention gave rise to its successful embedding in practice; and whether new testable propositions could be made about the factors that are likely to support the potential for embedding new interventions in the context of speech and language therapy. At the time when I undertook the analysis for this study, the NPM was in use, and the NPT was in its final development. Testing the applicability of the NPT and the generalisability of its explanatory power in understanding implementation and embedding of interventions within healthcare has been approached using a range of study methodologies and healthcare contexts [26,27], but so far, its applicability to the context of service delivery by Allied Health Professionals has not been tested. The study adopted a case study approach using qualitative data on a successfully embedded speech and language intervention to address research questions above. The third aim of the study will be addressed in the discussion to this paper.

## Methods

The study began with a search for a pediatric speech and language therapy intervention that was used in practice across the UK and was the topic of published qualitative research on the parents' and professionals' perspectives of the intervention. The pediatric speech and language therapy intervention that was commonly used the UK and had the most number of published studies of qualitative research as identified. The Hanen Parent Programme (HPP) originated in the US [28] and it has become embedded in practice throughout the UK during the past ten to fifteen years. The intervention uses an indirect method of therapy, which means that the practitioner works through another agent in order to achieve change in the child. In this case, the agent of therapy is the mother or caregiver. The practitioner uses video footage to help parents, who attend in groups, to see how they could adapt their own interaction to support the development of communication in their child. The communication targets can be verbal or non-verbal, making this a useful intervention for a wide range of children who present with different types of communication difficulties. Most speech and language therapy departments in the UK have a parent group based on the Hanen principles. The principles of the Hanen intervention are commonly found on all pre-registration speech and language therapy degree courses in the UK.

A literature search was conducted to find all research that had been published on the parental views of the HPP. In the first instance databases were searched using all search terms associated with the HPP (Hanen, Hanen Parent Programme, It Takes Two To Talk). This search identified approximately 20 papers. All these papers were downloaded and read in full to find all research that had included information on either the parental views of the intervention or the speech and language therapists' views on the intervention. There were five papers that presented data on the parents' or therapists' views. Three of these papers used semi-structured interviews or focus groups to elicit participants' views on the intervention. Two of the papers presented data from questionnaires that were used to elicit parental views of speech and language therapy. These studies were included because they explored parental views on direct (traditional) versus indirect (such as the HPP) approaches with children and families. The papers are summarised in Table 1.

**Table 1:** Studies included in analyses

Study	Participants	Measures and Analysis
Girolametto, Tannock and Siegel (1993)	Mothers who had taken part in a HPP N = 32	Likert satisfaction questionnaires with descriptive analysis Videotaped interaction of parent-child interaction with coding of behaviour
Glogowska and Campbell (2000)	Parents who had taken part in a RCT to evaluate traditional SLT intervention in pre-school children N = 16 selected respondents according to the logic of maximum variation	Semi-structured interviews framework analysis
Glogowska, Campbell, Peters, Roulstone and Enderby (2001)	Parents who had taken part in a RCT to evaluate traditional SLT intervention in pre-school children. N = 89	12-Item questionnaire with factor analysis (SLT frame of reference)
Baxendale, Frankham and Hesketh (2001)	Parents who had taken part in a controlled study to compare HPP with traditional clinic-based SLT N = 37 in total	Semi-structured interviews with parents Satisfaction questionnaires
Pennington and Thomson (2007)	SLTs who deliver the HPP in the UK N = 16	Focus Groups with SLTs Thematic analysis

### Defining method for secondary analysis

The first step in the analysis was to identify the important and recurrent themes that arose in the five studies in the review. The next step was to map the recurrent themes on to the constructs of the NPM [20]. The existing published research on the HPP did not contain data that was relevant to the two exogenous components of the NPM, but there were recurrent themes that mapped on to the two endogenous constructs of the model. The next step was to isolate all the quotations that were reported in the three papers that included data from semi-structured interviews of focus groups. All the direct quotations were taken out of the thematic context in which they had been grouped in the original research. They were read and then considered for inclusion into a construct map of the NPM endogenous factors.

The first endogenous process in the NPM concerns the professional-patient relations, the interpersonal context for normalisation, named Interaction Workability. The specific elements of Interaction Workability and the relationship between these elements are summarised in Table 2. According to May, an intervention that gets embedded in practice is likely to be one that allows flexible accomplishment of both congruence and disposal. The emphasis is on the flexibility needed for parties to combine their ideas and beliefs (congruence) and make them concrete in outcomes that are meaningful to both parties (disposal). According to the proposition in the model, the successfully embedded HPP should reveal evidence of flexible interpersonal work between practitioner and parent.

**Table 2:** Interactional workability, congruence gives rise to disposal

Congruence - bringing ideas together	Disposal - the outcome of combined thinking
Co-construction of core beliefs about the work →	Setting shared goals
Finding legitimacy in the outcomes of the work →	Establishing the meaning of the goals
Agreeing rules about the conduct of the working relationship →	Setting expectations about the outcomes of the work

The second endogenous process defined by May [20], named Relational Integration, covers the network of relations in which the clinical work is embedded. According to May, this network of relations is how the knowledge and practice of the intervention is defined and mediated. This is comprised of two dimensions, accountability and confidence. Accountability refers to internal network and has three components. These are: validity of the knowledge associated with the intervention, which includes ways in which disputes about that knowledge are minimised and the distribution of the knowledge within the hierarchies in the network; expertise, beliefs about the expertise entailed in the intervention; and dispersal, the distribution of knowledge and practice within the network. Confidence refers to the external network and has three components. These are: credibility, the development of a shared understanding of the credibility of the intervention, the ways in which disagreements about the intervention are handled, agreement about how credibility of the intervention should be measured; utility, beliefs about the source of knowledge and about the utility of those sources of knowledge; and expectations about the authority of the dispersion of knowledge in the external network. According to the proposition in the model, the successfully embedded HPP should reveal evidence of shared accountability and wide distribution of accountability across the agents involved in the intervention. The secondary analysis searched for evidence to test these two propositions using the direct quotations that were published in the original studies.

## Results

### Research question one

Are the definitions as described within the original version of the NPM applicable to a new data set based on a synthesis of qualitative research from previously published research on the HPP?

The findings of the synthesis of the qualitative research against the NPM propositions were checked by both of the NPM main authors (May and Finch) to search for inconsistencies or inaccuracies in the allocation of qualitative research to aspects of the model. No discrepancies were found. The allocation of direct quotations to the NPM was relative straightforward, and there were no discrepancies in the allocation between the first author of this paper and the NMP's main authors, however, it was important to consider the degree to which the NPM provided an inclusive framework for the main thematic findings in the original articles. The quotations as well as the main thematic findings from the original studies were used to populate the NPM framework (endogenous processes). The findings of the secondary data analysis was presented to academic speech and language therapists at Newcastle University who considered the findings to be congruent with their own experience of working with the HPP.

### Research question two

Can the application of the model uncover new understanding of how the interpersonal work done by the participants of the intervention gave rise to its successful embedding in practice? This was approached by testing the findings against the main propositions in the NPM on the endogenous factors of an intervention.

Is there evidence of flexible interpersonal work between practitioner and parent? The data in Table 3 show several areas of flexibility, and this is particularly evident in the parents. Parents start off expecting the child to be the focus of therapy (co-operation, legitimacy, and conduct), but the data on disposal (goals and meaning) show that parents have accepted that they are the legitimate target of therapy in the HPP. In focus groups with speech and language therapists, Pennington and Thompson [29] reported that the speech and language therapists valued how the parents had been able to adopt a totally different approach, they related this change in parents' communicative style positively, and they attributed the change to the content and delivery of the programme. The evidence of flexibility in practitioners is less noticeable, but there is evidence that they adjust the components of therapy according to parental feedback (see Goals). From the data in Table 3, it is evident that the speech and language therapists appear to carry the knowledge of the limits of the research evidence for the intervention with them. However, there is evidence in the data that the therapists focus on the theoretical principles that underpins the rationale for the intervention. They use these principles to theorise about change in the child. Furthermore, they assess outcomes of the therapy using primary data on the parent/child interaction. The data on conduct of both parent and practitioners show that both parties had similar expectations that the intervention would produce change, that the expert agent in this change would be the practitioner, and that the parent would follow the advice of the practitioner. It is possible that the flexibility in parents' perspective on their role in the intervention was facilitated by the explicit first-principle-theorising by the practitioners on how change will happen in the child as a result of changes made by the parents.

**Table 3:** Interaction workability qualitative data from parents' and practitioners' perspectives.

	Parent Perspective	SLT Perspective
**Congruence**		
Co-operation	Parents believe that the SLT will identify a problem in their child and treat it Parents expect to have their expectations met Parents are not sure how effective SLT intervention is Parents have hopes and fears about having their child's difficulties identified	SLTs believe they will locate problems in the parents and that they will treat those problems SLTs believe the intervention can be successful SLTs believe that the intervention is not successful for all participants SLTs believe that the intervention may highlight the chronicity of the child's difficulties for the parents
Legitimacy	Parents expect SLT to work on a 1:1 basis with their child Parents expect to follow advice from the SLT Parents expect this to lead to change in their child	SLTs believe that the parents are the legitimate focus of the intervention SLTs expect parents to follow advice from SLT and change their interaction with their child
Conduct	Parents expect to be able to have discussion Parents value partnership approach in relation to child's speech/language impairment Parents expect the SLT to help the child produce normal speech behaviours and they expect to be able to observe that happening in the SLT sessions	SLTs expect parents to follow their advice SLTs expect to see demonstrable change in the parent using video examples in the sessions
**Disposal**		
Goals	Parents accept that the goal of the intervention is related to their interaction strategies with their child	SLTs believe that HPP helps them set joint goals with parents SLTs adjust the components of the HPP intervention due to parental feedback/preference
Meaning	Parents accept that they are the focus of the intervention and can recognise change in their interaction and they attribute this change to the HPP	SLTs believe that the HPP will lead to predicted changes in the parent and they can theorise that this will impact positively on the child, but they know that this has not always been demonstrated in research studies
Outcomes	Parents would like to see outcomes in everyday activities and value an increase in normality in their child's behaviour	SLTs look for outcomes of the intervention in the verbal and non-verbal interaction between the parent and the child

A complex intervention is disposed to normalisation if it confers an interactional advantage in flexibly accomplishing congruence and disposal

Note HPP - Hanen Parent Programme

We might assume that the professional competency of speech and language therapists means that they are highly skilled in supporting the types of flexible co-construction that May says supports the normalisation of an intervention. If this is the case, then we might always expect to find evidence of flexible construction of agency in the context of speech and language therapy. Data from Baxendale *et al. *[8] suggest that this is not the case. They compared parental perspectives on the HPP with traditional clinic-based therapy following a randomised control trial of the two interventions. The expectations of all parents prior to speech and language therapy was that the therapy would be provided on a one-to-one basis with the practitioner providing the therapy in a clinic environment, and that the work would involve some direct elicitation procedures, such as helping the child imitate sounds or repeat sounds. The authors note that the parents who went on to receive the HPP found this expectation difficult to assimilate with reality of the indirect approach, 'But I was very much against it. I thought Eddie was going to be more like individual speech therapy sessions .... and I thought no it's Eddie that needs the speech therapy not us.' However, parents who were assigned to the HPP adopted the programme philosophy over the course of the intervention. Parents were positive about the indirect approach and, as we have seen, could attribute change in the child to their own intervention. In contrast, Baxendale *et al. *found that the parents who received the direct, traditional clinic-based therapy could state how they had changed their interaction, but did not see themselves as being responsible for outcomes. Therefore, it is not the case that practitioners are always successful in helping parents change their perspective on their role in the intervention. One conclusion from this analysis is that the HPP is an intervention that is particularly good at helping practitioners theorise about, discuss, and evaluate the mechanisms of change in the intervention.

Pennington and Thompson [29] also reported that the practitioners perceived increased confidence and empowerment in the parents who took part in the HPP. The practitioners in this study often attributed this change to the group delivery of the programme. However, practitioners also reported that they found the group work particularly difficult and that parents were apprehensive and insecure in the group. In congruence with this, it was often the group delivery aspect of the intervention that was dropped by the practitioners. The parents did not attribute any change in their own learning or feelings of confidence to other parents in the group. They often reported that they disliked the group delivery aspect of the programme. The practitioners attributed positive change in parents to other parent participants, even though this ran contrary to their own observations of how parent groups made the participants feel. In summary, the practitioners are aware that the intervention is about helping parents understand why they are targets of therapy; the practitioners are successful in doing this and they are aware of their success in this regard, but they do not attribute the changed state in parents (*i.e*., increased confidence) to their own interpersonal work within the intervention.

There was no evidence of flexibility in the area of outcomes of the intervention and how they were measured. There was an indication that the practitioners in Pennington and Thomson's study had ranked or analysed the pre-intervention communication style in the parents and were monitoring parents' progression. The primary outcome was change in parental communicative style, and the process was speech and language therapists' individual planning for each parent with a theory of what would bring about change in this primary outcome. The practitioners did not talk about how parents were active participants in the planning or measurement of their own outcome.

Is there evidence for flexibility in accountability and confidence in the intervention? The analysis on proposition is in Table 4. Despite the observation that parents want speech and language therapists to impart their specialist knowledge to them, the only evidence of any change in role in terms of accountability and confidence is found in the statement that after the intervention parents are willing to act as parent advocates for the intervention. Their continued requirement for speech and language therapy at the end of the intervention, the belief that the expertise in the intervention remains with the speech and language therapist, and the limited role that parents place in co-parents as co-learners shows that for parents the authority and expertise remains with the speech and language therapists. The analysis also showed up clear differences between the parent/practitioner views of the role of other staff in the National Health Service.

**Table 4:** Relational integration, qualitative data on parents' and practitioners' perspective

	Parent Perspective	SLT Perspective
**Accountability**		
Validity	Parents believe that the SLT has knowledge about normal development of speech/language and parents want SLTs to impart that knowledge to them	SLTs have knowledge that HPP is effective in changing parental communication style in the desired direction and this knowledge comes from reliable published sources and has been replicated SLTs are accredited as HPP practitioners
Expertise	Parents believe that SLTs can use their specialist knowledge to identify or confirm problems in their child They believe that the SLT is the best professional to do this Parents expect SLTs to provide them with practical advice to follow	SLTs that deliver HPP are experienced specialists in the field SLTs believe that parents' role is to follow their advice
Dispersal	Parents refer to the network of NHS practitioners as gatekeepers to their original attempts to gain access to SLT	SLTs build a network of knowledge on the HPP amongst their NHS colleagues
**Confidence**		
Credibility	Parents are willing to act as parent-advocates for the HPP	SLTs believe that parents are useful advocates of the HPP SLTs believe that other members of the NHS team can also be advocates SLTs believe that having HPP as part of the care-pathway for the child will support take-up of the intervention
Utility	Parents believe that the practice-based expertise of the HPP lies with the SLTs Parents do not speak about the role of other co-parents in relation to their own outcomes from the HPP	SLTs believe that parent-advocates and NHS-advocates are useful in convincing parents of the usefulness of the HPP - they help sell the HPP SLTs believe they are the best implementers of the HPP SLTs believe that the co-parents on the programme may support outcomes that they consider to be secondary in parents (increased confidence)
Authority	Parents continue to want SLT intervention for their child at the end of the programme.	SLTs are responsible for assessing outcome of the HPP Assessment is based on expert-knowledge of parent-child interaction which is located in the SLT

## Discussion

The small number of papers in this review, the absence of any previously published work on the perspective of the intervention from other stakeholders (*i.e*., other healthcare providers), and the absence of data that could be mapped on to the two exogenous processes of the NPM need to be considered prior to the discussion of the findings. In part, the small number of studies is representative of the status of qualitative research in the field of speech and language therapy. In a field that aligns itself culturally with the medical profession, the generation of evidence has relied primarily on quantitative research designs. There were no other interventions, which are widely used in routine paediatric speech and language therapy practice, that had a qualitative research literature suitable for this review. The absence of exploration of the wider stakeholders' views on the intervention and the absence of data that could have been mapped on to the exogenous factors in the qualitative research on the HPP is a product of the intentions of the primary investigators and their motivations for exploring the intervention. The production of the qualitative evidence base on the HPP was in large generated by academic speech and language therapists. The clinical academics' interest might explain the predominance of intervention-specific data from qualitative research. The absence of data on the exogenous constructs of the model does not imply that skill-set of workers and the organisational integration of the intervention is not relevant to the intervention. The HPP is an intervention that can only be delivered by qualified speech and language therapists, and is only delivered by therapists who attend special training in HPP and pass quality benchmarks for its use in clinical practice. Therefore, the intervention has a recognisable, well-bounded location within the speech and language therapy work force, which is suggestive of high potential towards embedding within routine practice (mapping on to the third construct of the NPM, that of skill-set workability). In relation to the integration within the wider organisation, to date, the work force of speech and language therapy within the wider healthcare work force could be described as having a well-defined and resourced responsibility for children with SLCN, and thus high potential for this intervention to be integrated in the institution's work force. We might find that the changes in healthcare commissioning and delivery in the UK and Bercow's recommendations for a wider distribution of the work to support children with SLCN (with the implication that this should extend well beyond speech and language therapists) that future qualitative intervention research in this field will probe the constructs that map on to the exogenous processes of the NPM. This remains to be seen.

Whilst the scope of the original research limits the strength to which this study can test the applicability of the NPM to speech and language therapy, the small number of papers does not appear to impact on the ability to address the research questions on the applicability of the endogenous constructs. The first testable component was to find out whether the definitions, as set out in the earlier version of NPT, the NPM, could be used as a basis for coding qualitative data on the successfully embedded HPP. The results from the reflexive work with the main authors of the NPM suggest that the qualitative data were accurately coded using the endogenous constructs within the NPM.

Having found that the constructs of the NPM provided usable coding definitions for the qualitative data, the second step was to find out whether the application of the model could uncover new understanding of how the interpersonal work done by the participants of the intervention gave rise to its successful embedding in practice. This review identified evidence of flexibility on the part of the parents and practitioners. Speech and language therapists adapted the intervention delivery on the basis of the users' perspective, and parents changed their beliefs about the legitimate targets of the intervention; they started with a belief that the practitioner should work directly with the child, but accepted that the practitioner would work directly with them in order to achieve change in the child. The concept of flexibility as a result of collaborative work between parents and professionals was not evident in any of the primary papers in the review. It appears that the application of the NPM has given rise to new way of thinking about the properties of the intervention in supporting the flexible collective work in the participants. In addition to the emergence of this new concept, the application of the NPM also opened up areas of incongruence between parents and practitioners' views on how the outcome of the intervention should be measured. Parents were more likely to rate success based on a positive change in their child's behaviour in a social context, whereas practitioners were likely to measure effectiveness by evaluating the specific linguistic behaviours of the parents. The proposition that arose from this review was that greater congruence between parents' and professionals' goals could be built into the intervention with the likelihood of enhancing parental satisfaction with intervention. Given the limited number of papers currently available in the context of the HPP, judgements about the full applicability of the NPT as an explanatory framework for work being conducted within this field remain tentative, however this early exploration of NPT within this context shows promise because it could help to shape the direction of the design and evaluation of complex interventions as the field develops.

### Evidence for testable propositions arising from this review?

The third aim for this study was to find out whether new testable propositions could be made about the factors that are likely to support the potential for embedding new interventions in the context of speech and language therapy. In this review, there is evidence of wide discrepancy in perspectives of the parent and practitioner and the network of other healthcare workers in relation to the intervention. The HPP has become successfully embedded in the UK; therefore, the finding of limited flexibility in accountability and confidence runs contrary to the predictions made by NPM. One reason could be that parents and practitioners are happy to have relatively passive parental roles within the clinical relationship and, therefore, no work on this aspect of the intervention is needed. In a qualitative study on parents' views of speech and language therapy in children with the most severe speech and language difficulties, Rannard [5] found that parents rate a partnership approach to their child's needs very positively. A partnership approach in educational provision was identified as a process that helped parents to change their initially negative opinion about special educational provision when they had a preference for mainstream education. We can learn from this that partnership working is valued by parents, they see it as a mechanism that will bring about change in their child, and there is evidence that team working by professionals can enable parents to feel more satisfied with provider-based recommendations for their child. Having an intervention with distributed accountability that is demonstrable to parents appears to result in more parental satisfaction. It seems that the issues of flexibility in accountability and distribution of the intervention rests with the healthcare provider side of the partnership.

When parents talk about getting a referral to speech and language therapy services in the UK, they frequently use words like 'fight' and 'battle' [1,5,6]. Rannard [5] sampled 40 families and found an average time lag of two years between the point when parents first had concerns about their child and the point of referral to a speech and language practitioner. Although some parents reported that they had delayed seeking help because they thought the child might grow out of the problem, in most other cases parents said that delayed referral was due to problems in getting the primary healthcare professional to refer to speech and language therapy. Like some parents, primary care workers might also delay referral because of their knowledge of wide variation in normal language development, they might think that the child will eventually catch up. Alternatively, primary care workers might delay referral due to more general beliefs about speech and language therapy services, such as optimal time for referral or the effectiveness of the intervention. Primary care workers might also delay referral in order to ration what is perceived to be a limited resource. These are speculations, and at the present time we have no data on which to build a proposition to explore the primary care workers' 'gate keeping' behaviour that parents find so frustrating. However, this review suggests that more work on the distribution of speech and language interventions in paediatrics might be one design component that, if improved, could give rise to more satisfaction amongst parents over the speech and language therapy interventions that they receive. Thus, parental satisfaction may not only rely on the interpersonal factors within the consultation that are considered to be related to reported outcomes in primary care, such as achieving therapeutic alliance and power sharing [30,31], rather the distribution of the intervention within the healthcare system should also be considered as a design factor, amenable to change, that could enhance parents' satisfaction with speech and language therapy.

This review suggests that the successful embedding of this intervention has been largely due to the nature of the interpersonal work that has been conducted within the practitioner/parent relationship. However, within this construct it was clear that improvements could be made within the intervention on the flexible accomplishment of outcomes between practitioners and parents. The outcomes are considered to be negotiated within the interpersonal construct and, given the relative strength in that area, the chances of improving outcome negotiation are probably quite high. If the practitioners and parents could establish meaningful parental outcomes from the intervention, this might also help them distribute the intervention within the wider healthcare networks--a construct where the intervention has relatively limited evidence of success. Intervention outcomes that are aligned to the real-world needs of the parents might provide data for non-specialists healthcare workers with information that they would see as valuable to the parents. This would be expected to enhance confidence in the intervention. Additionally, parents might take on a more active role in establishing the intervention within the network of health workers and become more active parent advocates for the intervention. We can see that parents attribute change to themselves as a result of the intervention, if they were able to explain how the changes they had made resulted in meaningful changes for their child this would likely result in compelling case-study evidence for other healthcare workers and other parents. Parents with this type of knowledge and power base might be able to act as a gateway to the intervention for families who practitioners have found 'hard to reach.' It would also provide practice-based evidence that therapists could use to increase their own confidence in an intervention that has a relatively limited research evidence base.

## Conclusion

This review demonstrates that the propositions within the NPM and NPT can be applied to non-medical interventions within health such as the interventions of the allied health professions. It could be used to help designers of intervention research, who themselves are not specialists in social theory, to explore the dynamic social processes that are associated with embedding research innovations in practice. At the current time, public dissatisfaction of the wider context of service delivery to children with speech language and communication needs is quite high. This review uncovered new knowledge of the internal components of the intervention that were likely to have led to the successful embedding of the HPP. It also found that the intervention itself was poorly disturbed within the wider health services. This raises a couple of questions. Are all speech and language interventions poorly diffused within the wider public services? If so, is the poor distribution of speech and language therapy within public services a cause of the more general public's dissatisfaction with speech and language therapy? I have proposed that working towards greater congruence in the meaning of the intervention (by working towards the family's desired outcomes) could lead to parents becoming greater advocates for the intervention which itself could lead to better diffusion within the healthcare system thus generating a more fertile context into which new innovation in speech and language therapy could be expected to take root. This proposition could be used in future design of interventions in speech and language therapy, and could also be tested in development work in pre-phase one trials of new interventions.

## Competing interests

The author declares that she has no competing interests. 

## Author's Information

DJ prepared this article whilst employed as an academic speech and language therapist in Speech and Language Sciences at Newcastle University, UK. During that time she was a collaborating member of the Institute of Health and Society and was mentored by Professor Carl May. DJ is now working as a translational scientist in child and family at the NIHR National Biomedical Research Unit in Hearing (National Institute of Health Research) at Nottingham University.
